# Lightweight YOLOv8-based real-time detection of pine wilt disease from drone imagery

**DOI:** 10.3389/fpls.2026.1854303

**Published:** 2026-06-11

**Authors:** Haojie Chai, Junyao Liu, Jingli Guo, Teng Zhang, Bingqian Li, Yuqing Xu, Yanyan Wang

**Affiliations:** College of Artificial Intelligence, Henan Institute of Science and Technology, Xinxiang, China

**Keywords:** attention mechanism, lightweight model, pine wilt disease, UAV remote sensing, YOLOv8, data augmentation

## Abstract

**Introduction:**

A core bottleneck of forestry remote sensing lies in accurate, real-time pine wilt disease monitoring on UAV-borne edge hardware, which suffers from constrained computing capacity and complicated field forest backgrounds. To fill this technical gap, we developed an ultra-lightweight real-time detection architecture named Edge-Forest YOLO in this work.

**Methods:**

Methods: Built upon the baseline YOLOv8n network, three targeted optimizations were embedded into the proposed model: (1) a domain-adaptive data augmentation workflow to mitigate poor generalization induced by variable illumination and uneven lesion sizes in complex woodland; (2) scale-aware asymmetric channel redistribution, cutting 37.5% shallow channels and expanding 75% deep channels to remove redundant spatial features and strengthen high-level pathological feature extraction; (3) Cross-layer ECA attention adopting 1D convolution to capture inter-channel correlation and concentrate on diseased regions with minimal computation overhead. All model validation was performed on the public high-resolution UAV pine wilt PDT dataset.

**Results:**

Edge-Forest YOLO only occupies 2.31 M storage with mAP@0.5 up to 92.7%. Its single-image inference costs 4.2 ms on regular computing equipment and runs at around 26 FPS on the Jetson Nano edge platform. Compared with YOLOv8s and customized YOLO-DP, our model cuts over half parameter quantity while retaining competitive detection precision.

**Discussion:**

The proposed lightweight detector supplies a low-power, practically deployable solution for on-board UAV real-time forest disease monitoring, supporting rapid in-field pine wilt diagnosis and facilitating scientific decision-making for forest health management and disease prevention.

## 1 Introduction

Pine Wilt Disease (PWD), caused by the pine wood nematode, is a devastating forest disease. Due to its short incubation period, high infectivity, and extremely high mortality rate, it has been metaphorically referred to as the “cancer” of pine trees, posing a severe threat to global forest ecosystems (Chen and Li, 2025; Wang and Zhang, 2025). Since its first detection in China in 1982, the disease has spread to 19 provinces (Ye, 2019; Chen and Li, 2025). Infected pine needles typically change from green to yellow-brown or reddish-brown within approximately 40 days, eventually leading to the death of the entire tree. This conspicuous visual symptom provides a critical spectral basis for rapid, large-scale disease monitoring using unmanned aerial vehicle (UAV) remote sensing (Espada et al., 2025; Wang and Zhang,, 2025).

Early PWD monitoring primarily relied on traditional image processing and shallow machine learning methods. For instance, Zhou et al. (2025) utilized UAV RGB imagery combined with multi-scale segmentation and support vector machines (SVM), integrating vegetation indices and texture features to achieve an 85.45% classification accuracy. Huang et al. (2021) applied early YOLO algorithms to preliminarily localize discolored trees, attaining an mAP of 89.7%. However, in real-world aerial images with complex forest backgrounds and variable target scales, traditional methods gradually revealed limitations in generalization and robustness against background interference. With the rapid development of deep learning, convolutional neural network (CNN)-based detection frameworks have demonstrated remarkable performance in improving accuracy. Xu et al. (2023) and Liang et al. (2025) incorporated attention modules and improved backbone networks, raising mAP to 91.5% and 94.21%, respectively. More recently, Feng et al. (2025) proposed FLMP-YOLOv8 and Shi et al. (2025b) explored the YOLOv11 architecture, leveraging multi-scale perception and dual-branch attention mechanisms to push mAP to 93.5% and 94.7%. However, models that pursue extreme accuracy are often accompanied by high computational costs, limiting inference speed on embedded devices and hindering real-time industrial inspection.

For complex scenarios involving close-proximity and cross-platform pest detection by UAVs, advanced detection architectures are continuously explored. For example, Yu et al. (2025) recently proposed CPD-YOLO, which cleverly integrates re-parameterized visual transformers with dynamic detection heads, achieving 90.42% mAP and 88.86% F1 score while supporting the development of mobile intelligent inspection applications. This represents a critical frontier paradigm for high-precision, close-range pest recognition in agriculture and forestry. Modern smart forestry imposes comprehensive and stringent engineering requirements on UAV monitoring. Under the severe constraints of edge device resources, Ren et al. (2020) highlighted the necessity of efficient deep learning scheduling strategies to reduce edge-end energy consumption by up to 28%, while Engwerda et al. (2023) emphasized the importance of image masking techniques for safe and reliable UAV monitoring. Furthermore, PWD monitoring is evolving toward multimodal and large-scale early-warning applications: from extracting subtle spectral-frequency features using discrete wavelet transforms (DWT) (Zhou and Yang, 2025) and hyperspectral data (DEIM) (Shen et al., 2025), to incorporating Transformer-based global modeling (Shi et al., 2025a) and multi-scale contextual mechanisms for pixel-level segmentation (Zhou et al., 2026); Ha et al. (2025) innovatively integrated single-tree detection results from deep learning into species distribution models (SDM), enabling outbreak risk prediction over areas of 500 km². All these advanced, cross-disciplinary ecological warning applications rely fundamentally on “highly efficient and reliable single-tree detection at the edge”.

Consequently, the conflict between “high accuracy” and “extreme lightweight” has become a core bottleneck constraining the practical implementation of these visions. To address this challenge, research on lightweight architectures has made significant progress in recent years. Chen et al (Chen et al., 2025). compressed YOLOv7 parameters to 65%; Chen et al (Chen et al., 2025). proposed PWD-YOLO-D and Ye et al. (2023) developed PWD-YOLO, reducing parameters to 2.3M and 1.09M, respectively, thereby substantially improving inference frame rates. Similarly, SLMW-Net (Yuan et al., 2025), LW-PWDNet (Hu and Wang, 2025), and models optimized via TRIZ theory (Hao, 2025) achieved rapid deployment at approximately 2M parameters. However, existing lightweight strategies mostly adopt simple uniform scaling or direct replacement with lightweight backbones. Such approaches inadequately consider the critical role of deep semantic information for pathological pattern recognition in forestry. When parameters are extremely compressed, indiscriminate pruning of deep-layer channels can irreversibly diminish the model’s ability to represent highly occluded or small-scale lesion features, leading to drastic accuracy degradation.

To break the inherent “accuracy-efficiency” dilemma of pathological detection on ultra-low computational edge devices, this study proposes an ultra-lightweight detection framework, Edge-Forest YOLO, tailored for low-power, field-based forestry monitoring. Addressing issues such as suboptimal channel allocation, weak deep semantic extraction, and high computational redundancy in existing lightweight models, we innovatively introduce a scale-aware asymmetric channel reallocation strategy coupled with cross-layer efficient channel attention. This approach preserves deep pathological semantic features while compressing computational overhead, ultimately constructing a lightweight detection model suitable for edge deployment. The framework aims to overcome real-world constraints such as poor real-time verification and high hardware deployment thresholds, providing a reliable technical reference for low-power smart forestry monitoring.

## 2 Experimental dataset and domain-adaptive augmentation strategy

### 2.1 PDT-LL UAV remote sensing dataset and challenging scenarios

This study utilizes the open-source high-resolution UAV dataset for pine forest pests and diseases target detection, referred to as the Pests and Diseases Tree dataset (PDT) (available at: https://github.com/RuiXing123/PDT_CWC_YOLO-DP) to conduct related experiments. According to the official documentation, PDT is the first high-resolution UAV image repository specifically collected for tree disease target detection in real forestry operational environments. Considering the computational and memory constraints of UAV edge devices, we selected a subset of the original images with a resolution of 640×640, denoted as PDT-LL, to serve as the benchmark dataset for this study.

**Figure 1:**
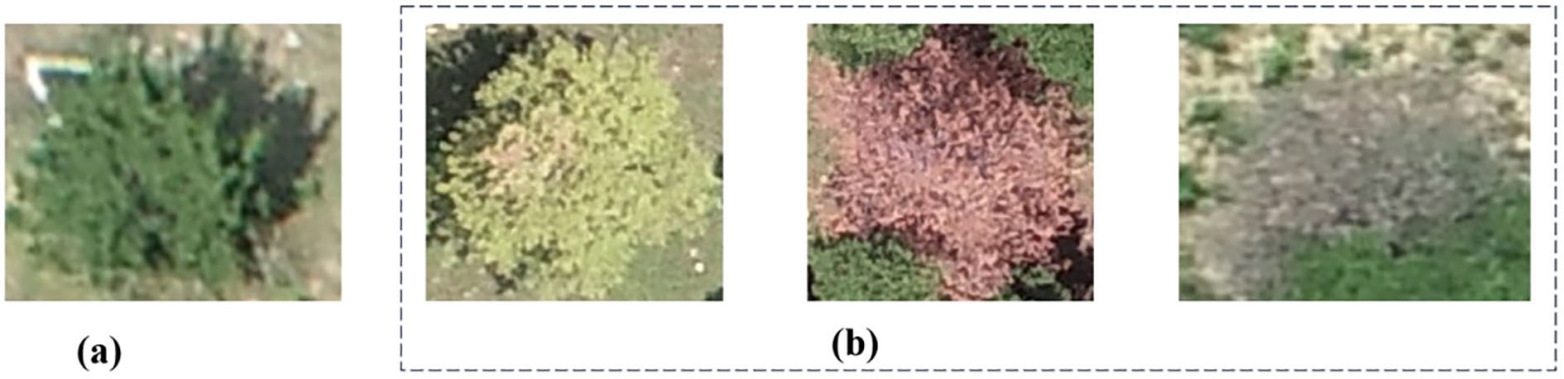
Comparison of healthy and diseased pine target samples in the PDT dataset. **(a)** healthy **(b)** unhealthy.

Visually, infected pine needles exhibit characteristic yellow-brown or reddish-brown coloration, which contrasts sharply with the green canopy of healthy trees. As shown in **Figure 1**, the benchmark visual features provided by the PDT dataset clearly highlight this distinction.

The dataset defines “unhealthy pine trees” as the sole target detection category and innovatively incorporates a large number of negative samples (untargeted images) containing exposed soil, healthy vegetation, and other strong background distractors, aiming to enhance the model’s robustness against false positives in real-world open environments. To ensure scientific rigor in model training and fairness in evaluation, this study strictly follows the official dataset split protocol. The detailed number of images and multi-scale instance distributions are summarized in **Table 1**.

**Table 1:** Dataset split and multi-scale instance distribution of PDT-LL.

Dataset split	Targeted	Untargeted	Total number of images	Total Instances	Small (<32×32)	Medium (32×32–96×96)	Large (>96×96)
Train	3166	1370	4536	90290	70418(78.0%)	16342(18.1%)	3530(3.9%)
Val	395	172	567	12523	9926(79.3%)	2165(17.3%)	432(3.4%)
Test	390	177	567	11494	8949(77.9%)	2095(18.2%)	450(3.9%)
Total	3951	1719	5670	114307	89293(78.1%)	20602(18.0%)	4412(3.9%)

**Figure 2:**
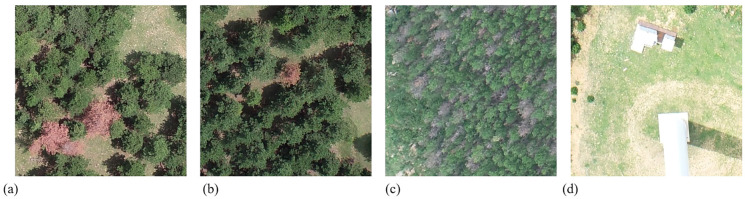
Typical challenging scenario examples of the UAV pine wilt disease dataset. **(a)** Large-scale aggregated disease, **(b)** Small-scare isolated disease, **(c)** High-density misxed canopy, **(d)** Strong background interference.

Although disease symptoms are relatively conspicuous at the single-tree or local view level (as shown in **Figure 1**), UAV-based aerial inspections often encounter severe visual interference and challenging environmental conditions. As illustrated in **Figure 2**, these are typical difficult scenarios captured during actual UAV operations.

Combining the complex habitat scenarios illustrated in **Figure 2** with the statistical data in **Table 1**, it is evident that the dataset exhibits an extremely high target density, with small- and medium-scale targets predominating. Such extreme multi-scale distributions, densely intertwined canopies (**Figure 2C**), and strong illumination and shadow interference (**Figure 2D**) can easily cause severe missed detections and feature collapse in traditional lightweight models during feature extraction. To address this, a customized domain-adaptive augmentation strategy was introduced during the data preprocessing stage in this study.

### 2.2 Domain-adaptive data augmentation pipeline

To address the natural challenges encountered during UAV operations, such as large target scale variations and densely intertwined forest canopies, and to prevent model overfitting without increasing edge-end inference computational overhead, this study designed and introduced a real-time, stochastic Domain-Adaptive Augmentation Pipeline during the training stage. The pipeline primarily employs the following three types of physically motivated transformations to specifically mitigate these visual challenges:

1. Geometric Spatial Transformations (Addressing Multi-Scale and Viewpoint Distortions):To enhance the model’s cross-scale detection capability for large-scale clustered lesions (**Figure 2A**) and small-scale isolated lesions (**Figure 2B**), a random affine transformation was applied, including rotation (± 8.0°), translation (± 0.1), scaling (0.6–1.4), and shearing (± 1.0). These parameters accurately simulate the dramatic changes in target scale and viewpoint caused by UAV hovering instability or varying flight altitudes. Additionally, horizontal random flipping was applied with a probability of 0.5, reflecting the prior assumption of symmetrical distribution in natural forest environments.
2. Color Space Perturbations (Addressing Sudden Illumination Changes and Strong Background Interference): As shown in **Figure 2D**, strong background distractions such as exposed soil and artificial structures, combined with extreme illumination variations during all-weather UAV operations, can easily obscure disease features. To mitigate this, random perturbations were performed in the HSV color space, with hue shifted by 0.015, saturation by 0.6, and value (brightness) by 0.3. This strategy forces the model to learn intrinsic lesion texture and relative color contrast features, rather than memorizing specific absolute colors, thereby improving robustness under harsh lighting conditions, including strong noon sunlight, localized shadows, or fog.
3. Complex Context Reconstruction (Addressing Dense Canopy Interweaving and Occlusion):To handle the extreme scenario of highly intertwined healthy and diseased needles (**Figure 2C**), Mosaic augmentation was applied with a high probability of 0.8. Inspired by Bochkovskiy et al. (2020) in YOLOv4, this strategy compels the model to extract discriminative features from fragmented contextual backgrounds, effectively alleviating the challenges posed by large target scale variation and dense canopy interweaving under UAV top-down views. Studies have shown that data augmentation strategies tailored to UAV-specific perspectives significantly enhance generalization robustness in complex remote sensing scenarios (Leng et al., 2023).

**Figure 3:**
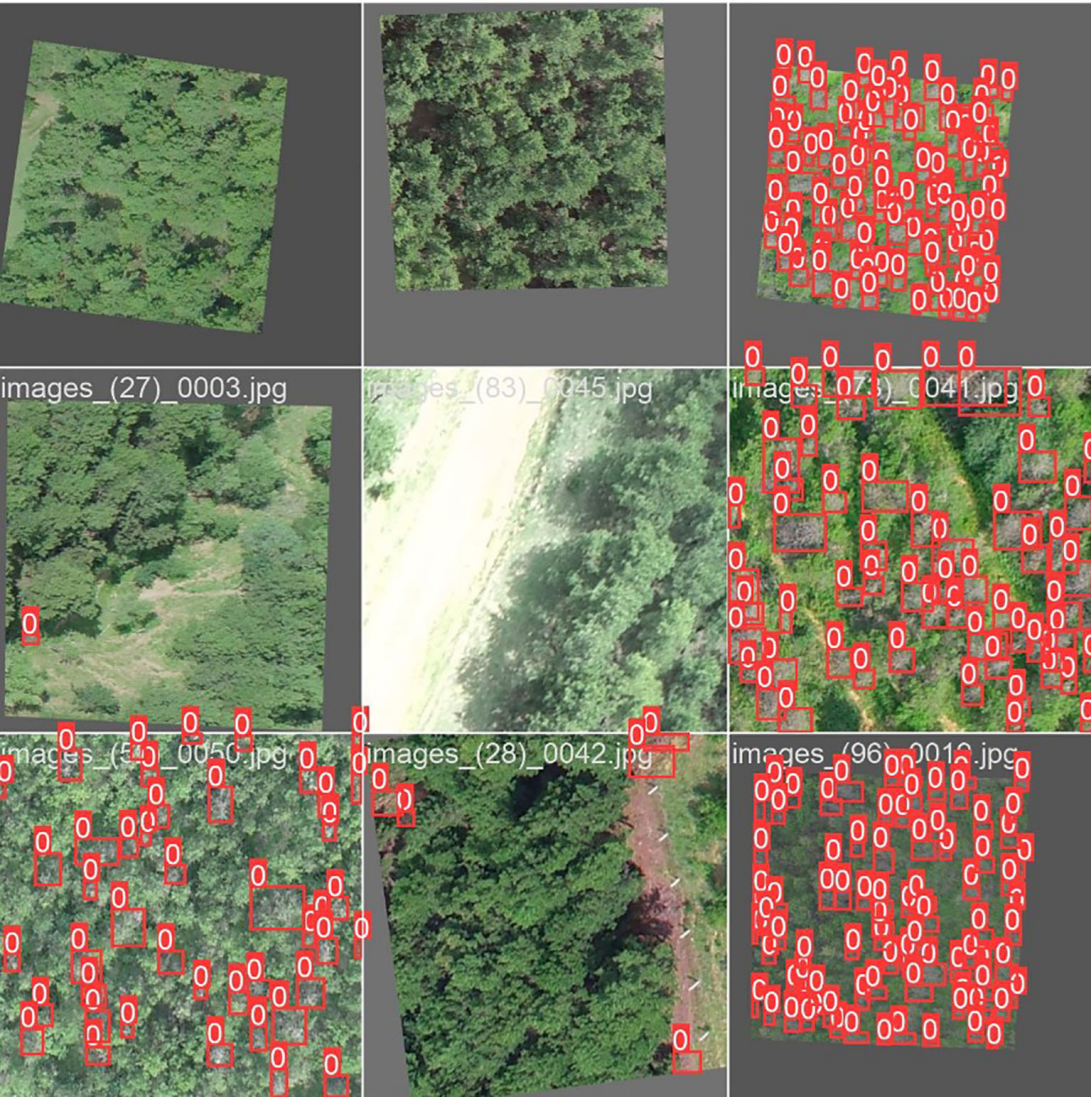
Example of domain-adaptive data augmentation effects during the training phase.

Specifically, four different training images are randomly cropped and concatenated, forcing the model to recognize lesions in more fragmented and disjointed contextual backgrounds, thereby improving detection of small or partially occluded targets. Additionally, the pipeline introduces randomly placed elliptical occlusion patches to simulate physical occlusion caused by overlapping branches or flying birds, ensuring that the model maintains high-confidence discriminative ability even when features are incomplete; the enhancement results are presented in **Figure 3**.

Through the aforementioned augmentation techniques, the feature richness of the training samples was increased by approximately fourfold compared to the original scenarios shown in **Figure 2**, effectively mitigating overfitting in ultra-lightweight models under small-sample conditions.

## 3 Task-aware lightweight detection method

### 3.1 Baseline model selection and feature analysis

In constructing an edge-based detection framework for pine wilt diseases, YOLOv8n was selected as the core algorithmic prototype. As a representative single-stage object detector, YOLOv8n achieves an excellent balance between inference efficiency and model size while maintaining high detection accuracy.

**Figure 4:**
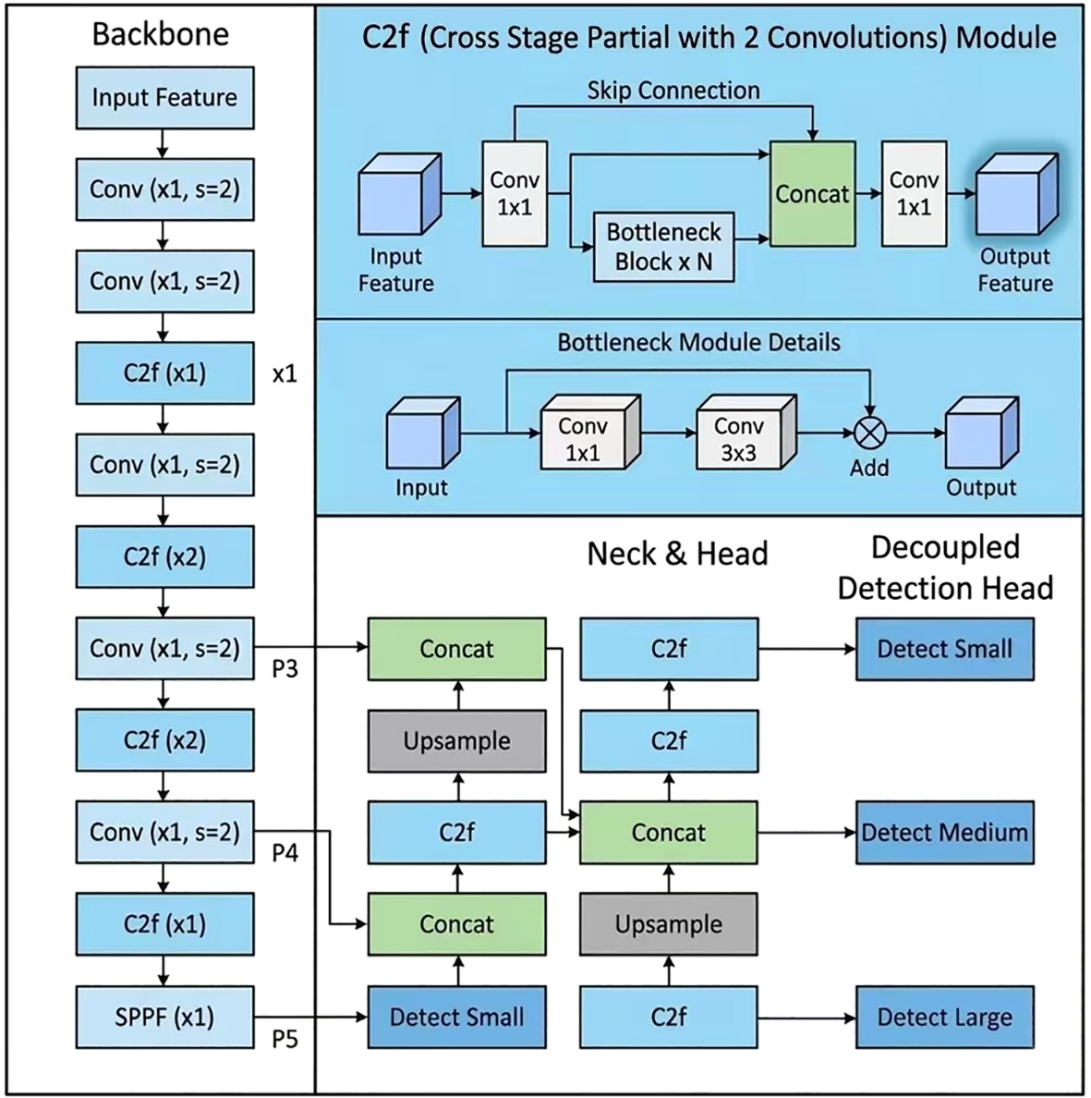
Schematic diagram of the original YOLOv8n network architecture.

As illustrated in **Figure 4**, the overall architecture of YOLOv8n consists of three main components: the backbone, the neck, and the head. The backbone employs a feature extraction structure with rich gradient flow, transforming the original image into multi-scale feature representations through multiple stages. This study primarily focuses on the terminal feature layers P3 (shallow), P4 (intermediate), and P5 (deep), which encode image information at different spatial resolutions and semantic levels. The neck adopts a PAN-FPN structure to achieve deep fusion of multi-scale features. Its core logic involves bidirectional propagation of deep semantic information and shallow texture details, enhancing the model’s sensitivity to targets of varying scales. The detection head features a decoupled design for classification and regression, enabling more precise bounding box predictions for lesions. Although YOLOv8n performs well on general datasets, experimental results indicate that its native model contains approximately 3.01M parameters with a single-frame inference time of 8.9 ms. For UAV edge devices, this standard channel configuration still incurs computational redundancy. Specifically, shallow features (P3) primarily contain detailed textures and exhibit substantial redundancy in dense forest backgrounds, whereas deep features (P5) still have potential to better capture disease-related semantic information. Therefore, using YOLOv8n as a baseline for targeted lightweighting and enhancement constitutes a scientifically grounded approach for achieving real-time and accurate UAV inspection.

### 3.2 Overall architecture of edge-forest YOLO

**Figure 5:**
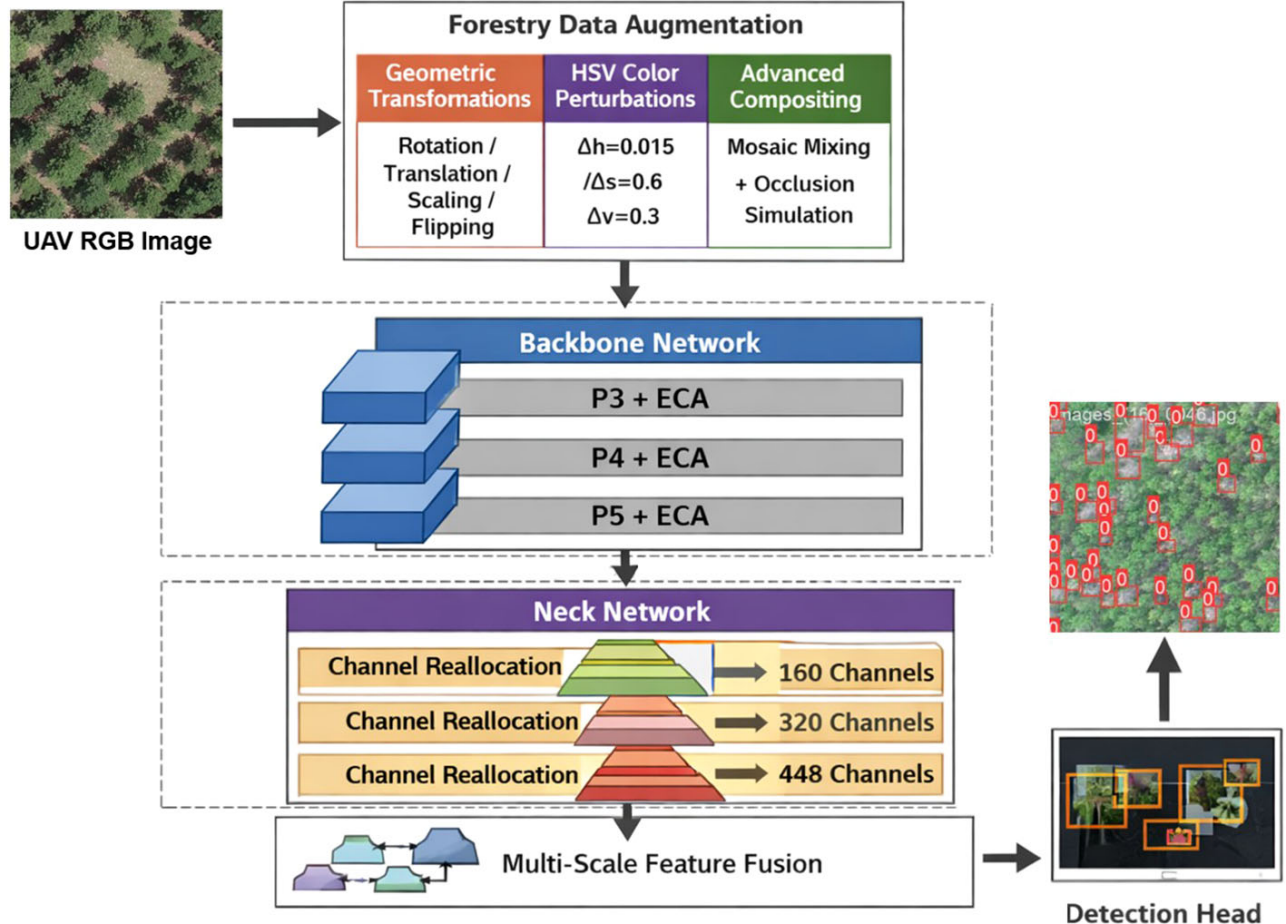
Overall architecture of edge-forest YOLO.

To address the triple challenges of severe environmental interference, imbalanced target scales, and extremely limited computational resources in pine wilt disease detection, this study presents a task-aware deep modification of YOLOv8n, resulting in the Edge-Forest YOLO ultra-lightweight detection framework. The full pipeline architecture of the model is illustrated in **Figure 5** and primarily consists of four components: a forestry-customized data augmentation pipeline, an improved backbone, an asymmetrically reallocated neck, and a decoupled detection head.

As illustrated in **Figure 5**, Edge-Forest YOLO follows a task-aware workflow of “domain-adaptive preprocessing → multi-scale feature focusing → lightweight feature fusion”:

1. Front-End Processing: The raw UAV-captured RGB images first pass through the Forestry Data Augmentation module. This module applies geometric transformations, HSV color space perturbations, Mosaic mixing, and simulated occlusion techniques to enhance the diversity and complexity of the input samples, forcing the model to learn more discriminative pathological features.
2. Feature Extraction: The preprocessed images are fed into the Backbone Network, where ECA attention modules are seamlessly integrated at the P3, P4, and P5 stages. This design allows the network to adaptively enhance channel weights associated with reddish-brown wilt features at different resolution levels while suppressing irrelevant green canopy background interference.
3. Feature Fusion and Channel Optimization: In the Neck Network, a core asymmetric channel reallocation strategy is implemented. By reconfiguring the channel numbers of the multi-scale feature pyramid to 160, 320, and 448 for P3, P4, and P5 respectively, the strategy reduces computational redundancy while improving the flow of deep semantic information.
4. Prediction Output: Finally, the optimized multi-scale feature maps enter the decoupled detection head, where post-processing operations such as non-maximum suppression (NMS) produce real-time disease detection results on mobile edge devices.

The architecture of Edge-Forest YOLO is designed to achieve an optimal balance between model accuracy and hardware efficiency. Leveraging the low-parameter advantage of ECA and the high computational efficiency of channel reallocation, the framework alleviates the traditional trade-off between “computational redundancy” and “accuracy degradation” encountered when deploying deep learning models on UAV edge devices, all while maintaining an ultra-lightweight model size of only 2.31 MB.

### 3.3 Scale-aware asymmetric channel reallocation

#### 3.3.1 Redundancy analysis of conventional uniform scaling mechanisms

In standard object detection architectures, such as the YOLOv8 series, the Feature Pyramid Network (FPN) typically employs a globally uniform width scaling factor for channel allocation. However, for UAV-based top-down detection of pine wilt disease, this uniform scaling mechanism overlooks the physical correlations between feature hierarchy and target characteristics.

According to feature representation theory, feature maps at different depths encode differentiated semantic information:

1. Shallow Features (P3, P4): These layers possess high spatial resolution and primarily encode high-frequency information such as edges and textures. In forest scenes, they are susceptible to spatial noise from complex undergrowth, shadows, and other clutter, resulting in significant computational redundancy.
2. Deep Features (P5): These layers have a large receptive field and capture high-level semantics representing the overall pathological patterns of mid- to late-stage disease. When the channel numbers of deep features are excessively compressed due to lightweighting strategies, the model may suffer from insufficient feature representation, particularly when detecting isolated lesions against complex backgrounds.

#### 3.3.2 Mathematical modeling of asymmetric channel reallocation

To optimize feature flow efficiency under limited computational constraints, this study proposes an asymmetric channel reallocation strategy, theoretically grounded in the Network Slimming framework introduced by Liu et al. (2017). The core idea is to identify and prune shallow channels with high spatial feature redundancy, thereby reallocating the limited computational resources toward deeper layers that contain high-level pathological semantics. As noted by Lin et al. (2020) in studies on convolutional neural network structural optimization, such structure-level lightweight design tailored to specific computational bottlenecks is a key approach to overcoming edge deployment barriers.

This strategy adjusts the number of channels *C_i_* in each layer of the Neck by defining a layer-wise scaling coefficient *α_i_* The adjusted channel numbers $C_{i}^{\prime }$ can be expressed as:


$$C_{i}^{'}=\left[\alpha_{i}\cdot C_{i}\right]$$

Based on the visual characteristics of pine tree diseases, a non-uniform (asymmetric) channel configuration scheme was formulated, as summarized in **Table 2**.

**Table 2:** Comparison of scale-aware channel reallocation schemes.

Feature hierarchy	Original number of channels	Improve the number of channels	Adjustment range	Core design basis
P3(80×80)	256	160	-37.5%	Streamline high-frequency texture redundancy and suppress forest background noise
P4(40×40)	512	320	-37.5%	Balancing spatial positioning accuracy and local semantic strength
P5(20×20)	256	448	+75%	Strengthen global pathological semantics and enhance complex scene recognition capability

#### 3.3.3 Trade-off analysis between computational efficiency and accuracy

In convolutional neural networks, the number of parameters is proportional to the square of the channel count. By applying a 37.5% substantial reduction to the P3 and P4 layers, the model significantly decreases computational overhead at the input stage, reducing the overall model size to 2.31M. Although the channel count of the P5 layer is increased by 75%, its feature map resolution is only 20×20, so the resulting increase in floating-point operations (FLOPs) is far smaller than the computational savings gained from pruning the shallow layers. Experimental results indicate that this “shallow redundancy pruning and deep semantic reallocation” strategy effectively compensates for potential feature collapse during the lightweighting process, allowing the model to maintain a practical mAP@0.5 of 92.7% despite the substantial reduction in model size.

### 3.4 Ultra-low overhead cross-layer ECA attention enhancement

#### 3.4.1 Theoretical evolution and limitations of attention mechanisms

Attention mechanisms mimic the selective attention characteristic of human vision, guiding models to prioritize processing of the most informative regions in the input. In lightweight network design, channel attention enhances feature representation by modeling inter-channel dependencies. Early architectures such as SENet achieved significant performance improvements; however, their use of fully connected (FC) layers to generate channel weights introduces severe dimensionality reduction, which not only disrupts direct channel dependencies but also incurs substantial computational overhead.

**Figure 6:**
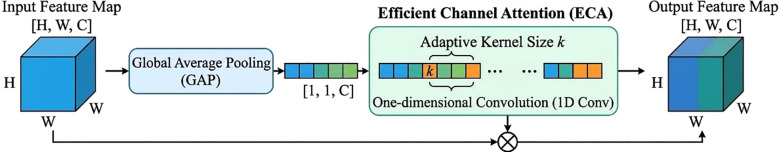
Schematic diagram of the ECA attention mechanism principle.

To achieve precise disease feature extraction under extremely limited computational budgets, this study incorporates the Efficient Channel Attention (ECA) module. The core innovation of ECA lies in eliminating the dimensionality reduction step of conventional attention mechanisms, replacing it with 1D convolution to enable local cross-channel interaction. This design overcomes the degradation of direct channel dependencies caused by the reduction operations in traditional attention mechanisms (e.g., SE-Net) (Wang et al., 2020).Through this non-reduction, locally recalibrated feature strategy, the model can adaptively “highlight” subtle textures in the reduced feature layers that correspond to reddish-brown lesions, significantly improving the signal-to-noise ratio for forestry disease monitoring. The detailed computational process of the module is illustrated in **Figure 6**.

#### 3.4.2 Algorithmic principle and mathematical formulation of the ECA module

As illustrated in **Figure 6**, given an input feature tensorX∈R^(H×W×C),the ECA module performs feature recalibration through the following three steps:

1. Global Spatial Information Compression: First, through a Global Average Pooling (GAP) layer, the spatial features of each channel are aggregated into a one-dimensional statistical descriptor g(X), generating a vector with the shape [1,1,C]. The calculation formula is:


$$g{\left(X\right)}_{c}=\frac{1}{H\times W}\sum_{i=1}^{H}\sum_{j=1}^{W}X_{c,i,j}\left[cite:62\right]$$

2. Adaptive Cross-Channel Local Interaction: A one-dimensional convolution with a kernel size of K is used to capture the interdependencies between neighboring channels. The kernel size K is adaptively and dynamically adjusted according to the number of channels C to ensure optimal interaction coverage at different hierarchical depths. The generated channel weight coefficient ω can be calculated by the following formula:

$\omega =\sigma \left(C1D_{k}\left(g\left(X\right)\right)\right)\left[cite:65\right]$

(where σ is the Sigmoid activation function).

3. Multiply the weight ω with the original feature map X channel by channel to output the enhanced feature map $\tilde{X}$:


$$\tilde{X}=\omega ⊗X\left[cite:68\right]$$

#### 3.4.3 Module collaboration and pathological feature compensation mechanism

In the proposed Edge-Forest YOLO framework, the ECA modules are strategically deployed at the terminal stages of the backbone (P3, P4, P5) as well as at the feature fusion nodes of the Neck. Their core scientific contribution lies in the strong synergistic interaction with the channel reallocation strategy:

Feature Flow Compensation: As described in Section 3.3, a 37.5% channel reduction was applied to the P3 and P4 layers to eliminate redundancy. With the introduction of the ECA modules, the model can adaptively “highlight” subtle textures and color features associated with reddish-brown needles within the pruned channels, compensating for potential sensitivity loss caused by channel reduction.

Background Noise Suppression: Forest backgrounds contain a large amount of visual noise from healthy tree crowns that resemble diseased trees. The ECA modules enhance channels critical to disease features while suppressing irrelevant background channels, significantly improving the signal-to-noise ratio in complex scenes. Without introducing additional computational overhead, this approach increases mAP@0.5 by approximately 2–3%.

## 4 Experimental design and results analysis

This chapter aims to systematically evaluate the effectiveness of the proposed Edge-Forest YOLO model for pine wilt disease detection through multi-dimensional experiments. The experimental design encompasses ablation studies, comparisons with state-of-the-art models, and visual interpretability analyses, assessing the model’s overall performance across three core dimensions: accuracy, robustness, and deployment efficiency.

### 4.1 Experimental setup and evaluation framework

#### 4.1.1 Experimental environment and hardware platform

To ensure the reproducibility and objectivity of the experimental results, all model training and testing were conducted under a consistent hardware environment.

Training Environment: A single NVIDIA GeForce RTX 3050 GPU was used to accelerate the training of deep learning models.

Software Framework: The models were implemented based on the PyTorch 2.3.1 deep learning framework.

Edge Testing Platform: To evaluate the real-time performance of the models in practical applications, an NVIDIA Jetson Nano embedded computing module was employed for benchmarking inference speed (FPS) on the edge device.

#### 4.1.2 Evaluation metrics

To comprehensively evaluate the performance of the Edge-Forest YOLO model for pine wilt disease detection, this study establishes an evaluation framework encompassing detection accuracy, model complexity, and inference efficiency.

##### 4.1.2.1 Accuracy evaluation metrics

We first introduce the basic concepts from the confusion matrix: True Positives (TP), False Positives (FP), and False Negatives (FN). Based on these, the following key metrics are defined:

Precision (P): Measures the proportion of actual positive samples among the samples predicted as positive by the model:


$$P=\frac{TP}{TP+FP}$$

Recall (R): Measures the proportion of actual positive samples that are correctly predicted by the model:


$$R=\frac{TP}{TP+FN}$$

Mean Average Precision (mAP): This is the most essential comprehensive metric in object detection. Firstly, the Average Precision (AP) for a single category is calculated, which is the area under the P-R curve:


$$AP=\int_{01}P\left(R\right)dR$$

Then, the mean of the APs for all categories (N) is taken to obtain the mAP. This study focuses on mAP@0.5 (at an IoU threshold of 0.5) and mAP@0.5:0.95 (the average over different IoU thresholds):


$$mAP=\frac{1}{N}\sum_{i=1}^{N}AP_{i}$$

##### 4.1.2.2 Efficiency and complexity metrics

To evaluate the practical deployability of the model on UAV edge devices, the following physical metrics are introduced:

Number of Parameters: Measures the static spatial complexity of the model and directly affects memory usage.

Inference Speed: Measures the model’s real-time processing capability on edge devices such as NVIDIA Jetson Nano, either by the time taken for single-frame inference (ms/frame) or by the frames transmitted per second (FPS).


$$FPS=\frac{1}{T_{inference}}$$

Among them, T_inference_ is the average total time required to process a single image.

#### 4.1.3 Implementation details and training strategies

All model variants followed a consistent training strategy to eliminate random errors. Notably, although the original PDT-LL dataset images have a resolution of 640×640, to balance the computational cost and memory constraints of edge devices, the input images were uniformly and dynamically resized to 512×512 using letterbox padding with bilinear interpolation during training, validation (testing), and final edge inference. The specific hyperparameter configurations are summarized in **Table 3**.

**Table 3:** Training hyperparameter settings.

Configuration item	Specific settings
Input Configuration	Input image resolution: 512×512 (dynamically scaled from the original 640×640); Batch size: 12
Optimizer plan	Stochastic Gradient Descent (SGD) with momentum; momentum factor: 0.937; weight decay: 5e-4
Learning Rate Scheduling	Initial learning rate: 0.01; Scheduling algorithm: Cosine Annealing; Warm-up phase: 3 cycles of linear warm-up
Number of iterations	Training epochs: 100; Model selection: save the checkpoint with the highest mAP@0.5 on the validation set

### 4.2 Ablation study analysis

To rigorously evaluate the individual contributions and synergistic effects of the three core modules—Domain-Adaptive Augmentation (DA), Channel Reallocation (CR), and Cross-Layer Efficient Channel Attention (ECA)—this study conducted a full-factor, decoupled ablation study using YOLOv8n as the baseline. All experiments were performed under the same hardware environment and hyperparameter settings, and the quantitative results are summarized in **Table 4**.

**Table 4:** Ablation experiment results.

Model name	Data augmentation	Channel Redistribution	ECA attention mechanism	mAP@0.5	mAP@0.5:0.95	Number of parameters (M)	Inference speed (ms)
A	×	×	×	**0.933**	0.662	3.01	8.9
B	✓	×	×	0.938	0.658	3.01	8.9
C	×	✓	×	0.908	0.601	2.30	4.1
D	×	×	✓	0.932	0.653	3.02	9.0
E	✓	✓	×	0.918	0.625	2.30	4.1
F	✓	×	✓	0.931	0.653	3.02	9.0
G	×	✓	✓	0.928	0.645	2.31	4.2
H	✓	✓	✓	0.927	0.643	2.31	4.2

#### 4.2.1 Lightweight gains and feature degradation under structural reconfiguration

Analysis of the data in **Table 4** for Model A and Model C shows that, after introducing the Channel Reallocation (CR) strategy, the model’s parameter count dropped sharply from 3.01M to 2.30M (a reduction of approximately 23.6%), while the single-frame inference speed on the Jetson Nano edge device increased significantly from 8.9 ms to 4.1 ms. This demonstrates that precisely pruning redundant feature channels in the network can substantially enhance computational efficiency for UAV edge inference. However, rigid channel compression inevitably weakens the model’s capacity to represent fine-grained disease features, resulting in a substantial drop in mAP@0.5 for Model C to 0.908.

#### 4.2.2 Precise compensation via attention mechanism

To compensate for the accuracy loss caused by channel reduction, this study introduces the ultra-lightweight cross-layer ECA attention mechanism. Comparing Model C with Model G reveals that, while maintaining the lightweight architecture, the ECA module introduces only minimal computational overhead (parameter increase of 0.01M, inference time increase of 0.1 ms), yet successfully boosts mAP@0.5 from 0.908 to 0.928. This result demonstrates that cross-channel interaction effectively suppresses background noise, refocusing the model on the characteristic features of reddish-brown diseased pine needles, thereby achieving precision “compensation” with negligible overhead. Furthermore, comparing Model A and Model D (mAP 0.933 vs. 0.932) indicates that in over-parameterized networks without channel pruning, the ECA module alone provides limited gain. In contrast, when channels are severely compressed (Model G), the critical role of ECA is fully realized, further highlighting the necessity of combining CR and ECA modules.

#### 4.2.3 Comprehensive performance evaluation and pareto optimality

Domain-Adaptive Augmentation (DA), as a purely input-side, training-stage preprocessing strategy, enhances the model’s feature learning capacity and robustness without altering the network structure or increasing parameters or inference overhead. As shown in **Table 4**, four pairs of “with/without data augmentation” experiments (A vs B, C vs E, D vs F, G vs H) consistently validate this: all combinations maintain identical parameter counts and inference speeds, while yielding an average mAP@0.5 improvement of 0.003, demonstrating the efficiency of the data augmentation strategy.

For the final Model H, which integrates all three major improvements, the mAP@0.5 shows a slight difference of 0.001 compared with Model G. This minor fluctuation falls within the normal random variation range of deep learning object detection experiments and does not indicate a true performance degradation. Theoretically, the forest scene-adaptive augmentation pipeline (including random rotations, brightness perturbations, and local occlusion simulations) encourages the model to learn more generalizable pathological features rather than overfitting specific validation samples. Even with minimal validation accuracy fluctuations, the resultant improvement in robustness under complex real-world conditions has significant practical engineering value for UAV deployment scenarios.

#### 4.2.4 Qualitative P-R curve comparison and robustness analysis

**Figure 7:**
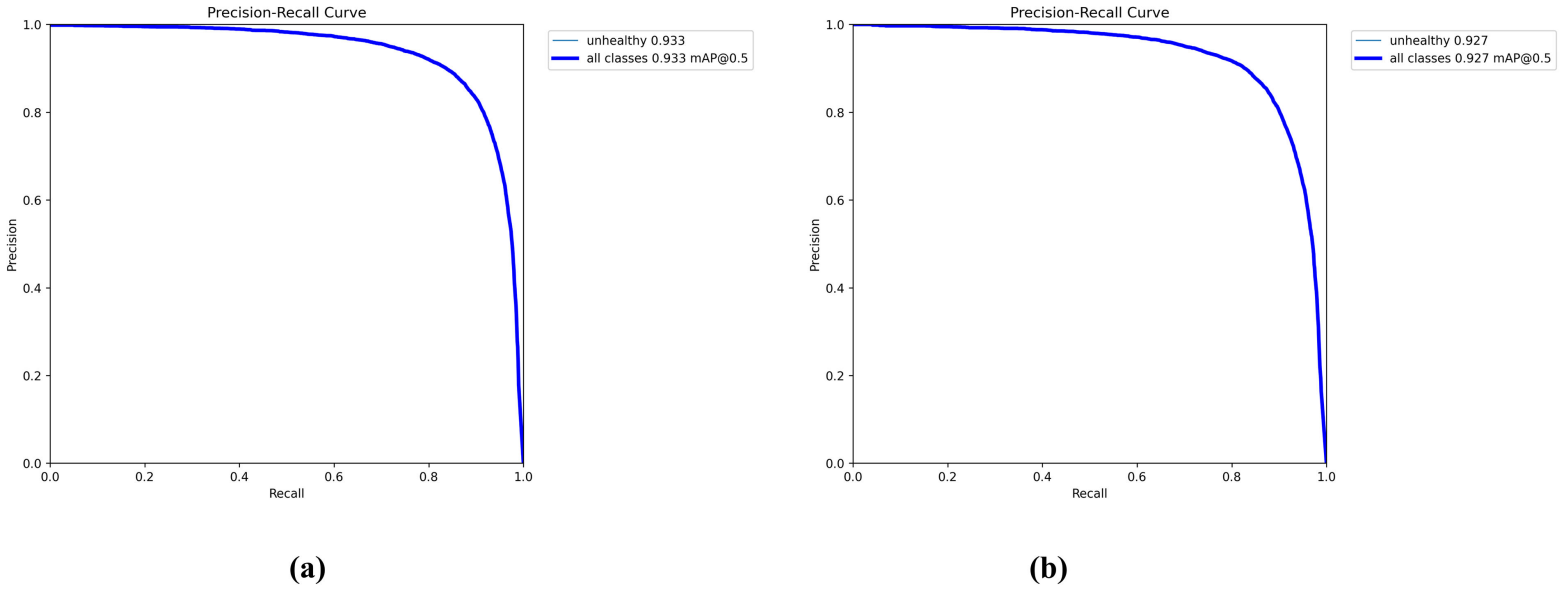
Comparison of P-R curve distributions for each model variant **(a)** YOLOv8n **(b)** edge-forest YOLO.

To further evaluate the effectiveness of the model improvements from a dynamic classification trade-off perspective, this study compares the Precision-Recall (P-R) curves of the baseline model and Edge-Forest YOLO on the validation set, as shown in **Figure 7**.

From a qualitative analysis of the curve shapes, the native YOLOv8n (Model A) demonstrates excellent feature-capturing capability, with an area under the curve (AUC) across all classes corresponding to an mAP@0.5 of 0.933. In comparison, the proposed Edge-Forest YOLO, despite undergoing Channel Reallocation (CR) that reduced its parameter count by 23.3% (down to 2.31M) and increased inference speed to 4.2 ms, maintains a robust mAP@0.5 of 0.927.

A careful comparison of **Figures 7A, B** shows that the P-R curve of the improved model preserves high precision at low recall regions, comparable to the baseline model. Moreover, during the decay phase as recall approaches higher thresholds, the curve remains smooth, without significant performance drops caused by network lightweighting. This provides direct evidence that the cross-layer ECA attention mechanism successfully compensates for feature loss due to channel pruning, enabling the model to accurately focus on characteristic reddish-brown diseased pine needles even for low-confidence candidate boxes, while effectively suppressing false positives caused by complex forest backgrounds. The final visualizations and quantitative results indicate that, under the models and hardware conditions compared in this study, Edge-Forest YOLO achieves a near-Pareto optimal trade-off between accuracy and computational cost.

#### 4.2.5 Training convergence and robustness analysis

**Figure 8:**
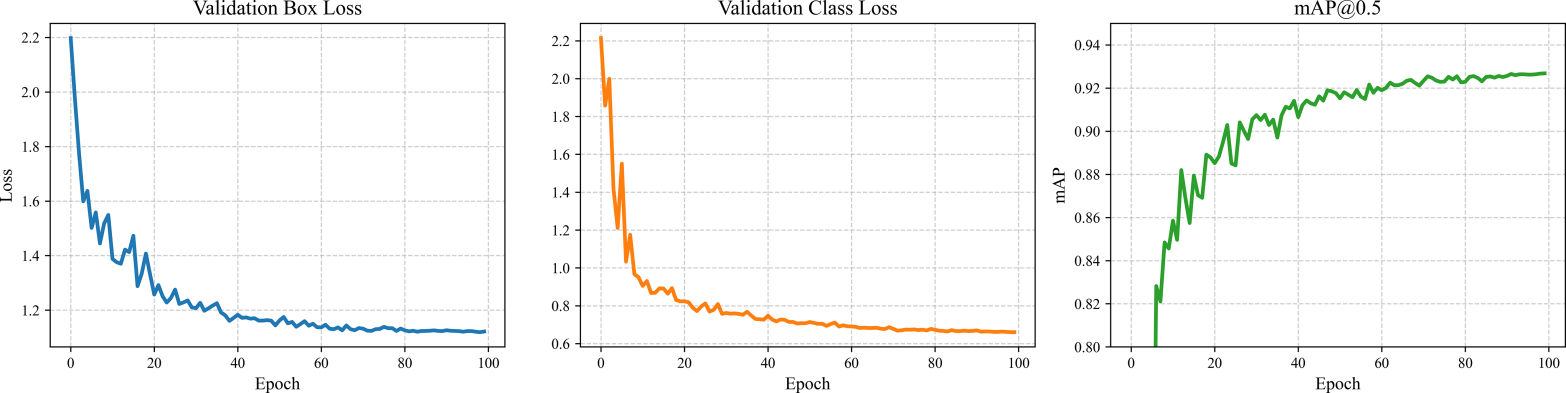
Loss and accuracy convergence curves of the edge-forest YOLO model training process.

To further demonstrate the training stability and anti-overfitting capability of the proposed architecture from a dynamic network optimization perspective, **Figure 8** illustrates the convergence curves of the final model Edge-Forest YOLO over 100 training epochs. The curves depict the validation box regression loss, classification loss, and the core evaluation metric mAP@0.5 on the validation set.

From the mAP@0.5 curve in **Figure 8** (right), it can be observed that the model’s accuracy improves rapidly during the early training stage (0–20 epochs), demonstrating efficient learning. This strong early convergence behavior provides intuitive evidence that the proposed non-symmetric channel reallocation strategy and cross-layer ECA attention mechanism form an effective low-level synergy, successfully unblocking the gradient flow of critical pathological features across shallow and deep network layers. Consequently, the model can quickly focus on disease regions even at the initial stage of training.

During the mid-to-late training phase, with the adoption of the cosine annealing learning rate schedule, the network gradually enters a smooth optimization saturation period, ultimately stabilizing mAP@0.5 at approximately 0.927. Notably, the first two loss curves in **Figure 8** (left and middle) indicate that throughout the 100 training epochs, both bounding box loss and classification loss decrease smoothly and monotonically, without oscillations or any signs of overfitting caused by rebound errors on the validation set.

The stable convergence characteristics demonstrate the effective role of the domain-adaptive data augmentation (DA) at the input stage. Even under extreme compression of network parameters to 2.31M, intensive geometric transformations and color perturbations help alleviate overfitting to simple forest background noise. Training and testing under challenging scenarios further indicate that the design of Edge-Forest YOLO enhances the model’s robustness against visual interference, allowing it to maintain strong generalization performance in complex UAV inspection environments.

### 4.3 Comparative experiments

To objectively and comprehensively evaluate the overall competitiveness of the proposed Edge-Forest YOLO in complex forestry inspection tasks, this section constructs two sets of comparative experiments based on the official test benchmark of the PDT-LL open-source dataset: one comprising different versions of YOLO models, and the other including mainstream non-YOLO detectors.

**Table 5** presents the comparative performance of mainstream non-YOLO detectors on the PDT-LL dataset. The results demonstrate that the proposed Edge-Forest YOLO achieves superior overall efficiency. Specifically, Faster R-CNN and YOLOv8n slightly outperform Edge-Forest YOLO in terms of mAP@0.5 and mAP@0.5:0.95, achieving marginally higher values than 0.927 and 0.640, respectively. However, both models incur significantly higher parameter counts, computational costs, and inference latency compared to the proposed framework.

**Table 5:** Comprehensive performance comparison of non-YOLO mainstream models on the PDT-LL dataset.

Comparison model	mAP@0.5	mAP@0.5:0.95	Number of parameters (M)	Computational load(GFLOPs)	Inference delay (ms)
YOLOv8n	0.938	0.661	3.01	12.03	8.2
SSD	0.756	0.583	23.75	30.53	17.31
Faster-R-CNN	0.945	0.676	28.28	227.82	50.08
Nanodet-plus	0.734	0.569	2.40	0.86	16.48
Edge-Forest YOLO(本文)	0.927	0.640	2.31	4.9	4.2

The comparison with mainstream object detection models confirms the advanced trade-off between accuracy and speed achieved by the improved YOLO-based architecture. To further substantiate the advantages of the proposed method within the YOLO family, and to rule out potential bias caused by baseline selection, a detailed intra-family comparison against other YOLO versions (e.g., YOLOv5, YOLOv7, YOLO26) is warranted. This enables a more comprehensive assessment of the proposed model’s performance relative to similar frameworks.

As shown in **Table 6**, the proposed Edge-Forest YOLO achieves an effective balance between detection accuracy and computational cost in UAV edge computing scenarios. Specifically, Edge-Forest YOLO attains mAP@0.5 and mAP@0.5:0.95 of 0.927 and 0.640, respectively, which is slightly less than the highest mAP@0.5 of the state-of-the-art YOLO-DP on this dataset (by less than 2%) and marginally lower than the mAP@0.5:0.95 of the classical lightweight model YOLOv8s (by less than 5%). However, its parameter count demonstrates a remarkable advantage, at only 2.31 M, representing a reduction of approximately 56% compared to YOLO-DP (5.2#x202F;M) and about 79% compared to YOLOv8s (11.1 M). In terms of computational cost, Edge-Forest YOLO requires only 4.9 GFLOPs, approximately 2/5 of YOLO-DP and 1/5 of YOLOv8s. While the YOLO26 model has a comparable parameter count and slightly lower FLOPs, its inference latency reaches 10.78 ms per frame—more than double that of the proposed model.

**Table 6:** Comprehensive performance comparison of different YOLO versions on the PDT-LL dataset.

Comparison model	mAP@0.5	mAP@0.5:0.95	Number of parameters (M)	Computational load(GFLOPs)	Inference delay (ms)
YOLOv5s_7.0	0.942	0.670	7.00	16.0	10.8
YOLOv7	0.901	0.555	37.20	105.1	31.3
YOLOv8s	0.940	0.679	11.10	28.6	16.7
YOLO-DP	**0.945**	0.675	5.20	11.7	9.2
YOLOv10-n	0.930	0.655	2.30	6.7	6.5
YOLO26	0.928	0.650	2.50	2.87	10.78
Edge-Forest YOLO(本文)	0.927	0.640	2.31	4.9	4.2

**Figure 9:**
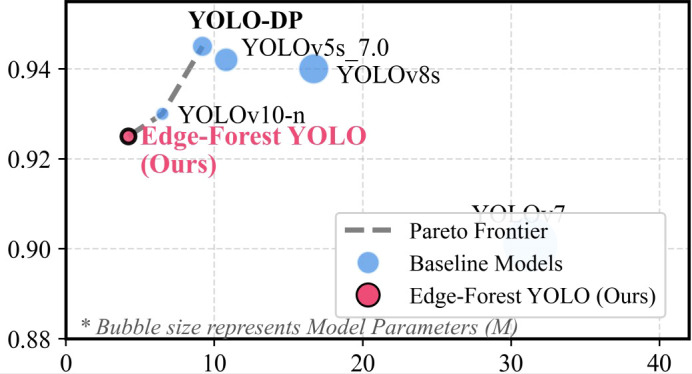
Comparison of the accuracy-latency Pareto fronts of different object detection models.

To further intuitively illustrate the trade-off between lightweight deployment and detection accuracy shown in **Table 6**, the core evaluation metrics of various detection architectures were visualized as a Precision-Latency Pareto frontier bubble chart (**Figure 9**). In this coordinate system, the x-axis represents single-frame inference latency (smaller is better), the y-axis represents mean Average Precision (mAP@0.5, larger is better), and the bubble size corresponds proportionally to the static parameter count of each model.

As depicted in **Figure 9**, traditional heavyweight networks, due to their large parameter size and high inference latency, are typically located in the upper-right region of the Pareto chart (higher latency/parameter count, higher accuracy). In contrast, the proposed Edge-Forest YOLO occupies the far-left region of the frontier, representing minimal latency and lowest parameter count. Compared with mainstream lightweight baselines, Edge-Forest YOLO significantly accelerates inference and substantially reduces model size while only sacrificing a marginal amount of detection accuracy, thereby achieving a Pareto-optimal balance for UAV edge deployment scenarios.

### 4.4 Analysis of computational performance and deployment feasibility

As shown in **Table 7**, the proposed Edge-Forest YOLO model effectively addresses the core conflict between high-precision detection and the low computational capacity of edge devices in UAV-based monitoring of pine wilt disease. The model achieves an industrial-level detection precision of 0.927 mAP@0.5 while reducing the number of parameters and computational complexity by 55.6% and 58.1%, respectively, compared with the state-of-the-art YOLO-DP model. On the Jetson Nano edge device, the actual measured inference latency is approximately 38.4 ms per frame, corresponding to approximately 26 FPS real-time performance, providing a solution that simultaneously satisfies accuracy and real-time requirements, thus occupying the Pareto-optimal region in the accuracy-efficiency trade-off. Furthermore, its ultra-lightweight architecture significantly reduces training and deployment costs, offering a cost-effective technical pathway for large-scale real-time UAV-based forest pest and disease monitoring.

**Table 7:** Computational efficiency and deployment feasibility.

Model	Parameters (M)	FLOPs (G)	Inference time per frame on RTX 4090 cloud server (ms)	Inference time per frame on NVIDIA RTX 3050 (ms)	Inference time per frame on NVIDIA Jetson nano (ms)	mAP@0.5
YOLOv8n	3.01	12.0	6.07	8.50	55.75	0.938
YOLO-DP	5.20	11.7	10.20	15.30	145.60	0.945
Edge-Forest YOLO	2.31	4.9	4.98	6.01	38.40	0.927

### 4.5 Visualization and analysis

To further validate the performance advantages of Edge-Forest YOLO from both qualitative and quantitative perspectives, this section presents an in-depth visualization analysis of detection results on the validation set. Error distributions are quantified using the confusion matrix, and representative inference cases in typical forest scenes are examined to assess the model’s robustness in complex environments.

#### 4.5.1 Confusion matrix and classification reliability assessment

**Figure 10:**
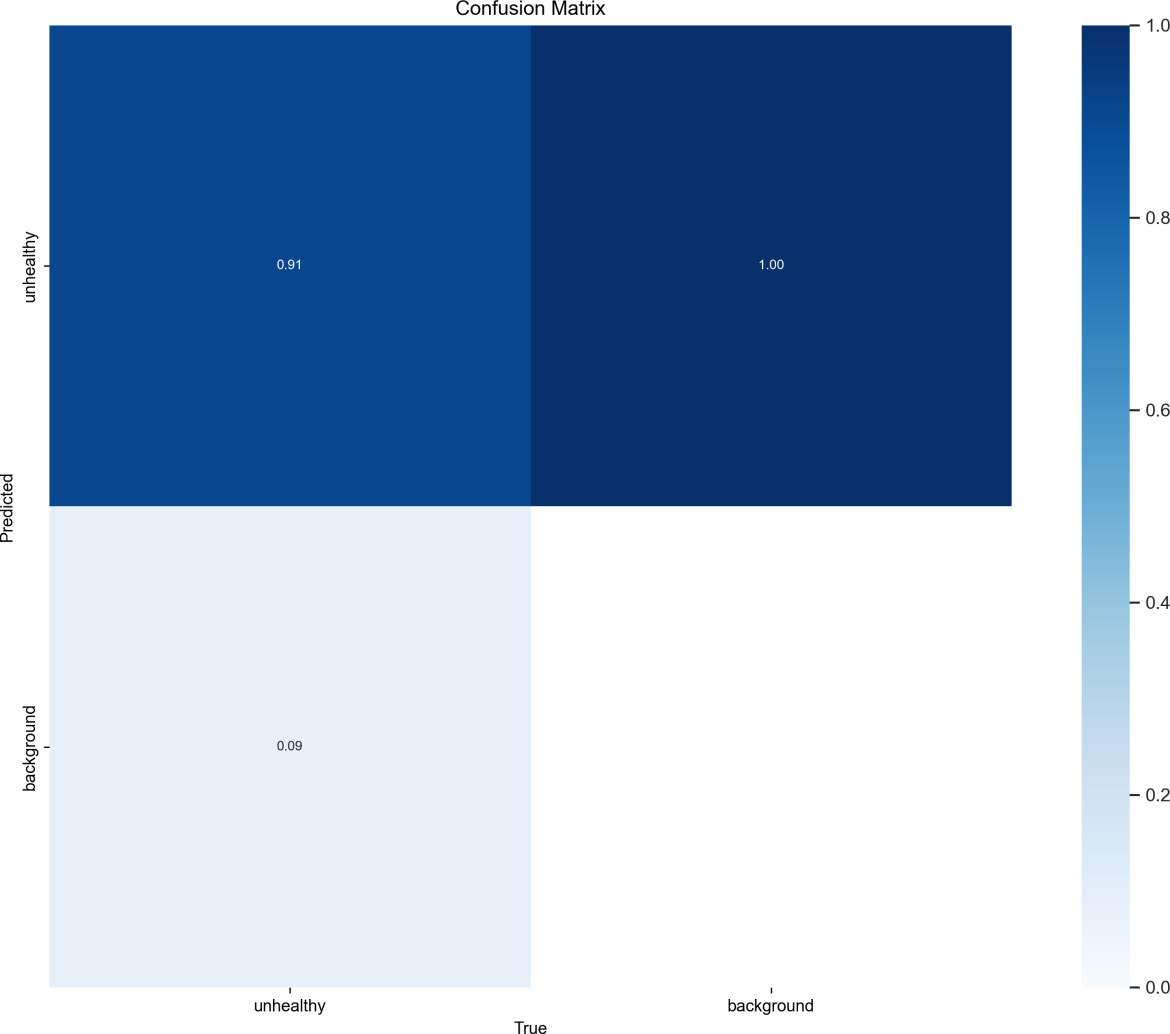
Confusion matrix.

The confusion matrix serves as a key tool for quantifying model classification biases. As shown in **Figure 10**, Edge-Forest YOLO demonstrates high discriminative reliability between background and disease (unhealthy) targets:

Extremely low false alarm rate: The matrix indicates that the proportion of background incorrectly classified as diseased is 0.00, yielding a background classification accuracy of 1.00. This statistically validates the physical significance of the ECA attention mechanism: through localized cross-channel interactions, the model effectively suppresses visual interference from healthy tree crowns and ground surfaces during feature extraction, concentrating decision weights on pathological features.

Stable disease detection capability: The classification accuracy for the unhealthy category remains high at 0.91. This demonstrates that even after compressing the model parameters to 2.31M, Edge-Forest YOLO retains strong lesion perception, effectively supporting UAV-based high-intensity forest inspection tasks.

#### 4.5.2 Detection performance in representative inspection scenarios

**Figure 11:**
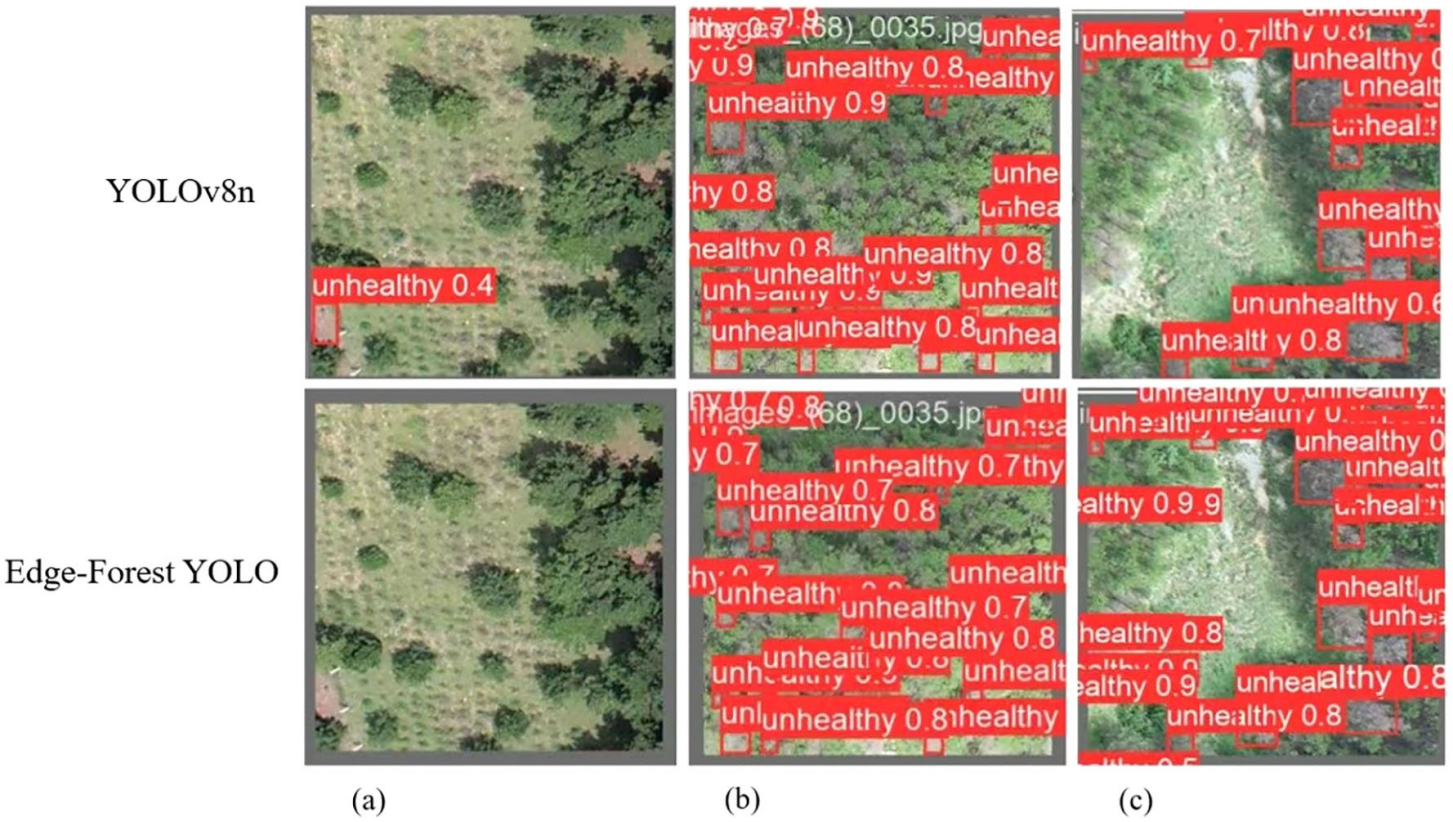
Visualization of detection results in typical inspection scenarios. **(a)** Sparse targets with strong background, **(b)** Dense and overlapping canopies, **(c)** Multi-scale targets at image edges.

To visually validate the performance gains of the proposed model in real-world complex environments, this study selected highly representative multi-scale and high-interference scenes from the validation set. The inference results of Edge-Forest YOLO were compared with the baseline YOLOv8n through multi-dimensional visualization, as illustrated in **Figure 11**.

Although the quantitative evaluation indicated a marginal compromise in global mean average precision to achieve the extremely lightweight 2.31M parameter deployment, the qualitative results in realistic forest scenarios demonstrate that Edge-Forest YOLO exhibits superior robustness against background interference and enhanced discriminative reliability compared to the baseline. Specifically, in sparse and high-interference scenarios (left column of **Figure 11**), the native YOLOv8n, constrained by excessive feature redundancy from large channel counts, misclassified areas of exposed soil or healthy vegetation in the lower-left corner as diseased. In contrast, Edge-Forest YOLO, benefiting from the cross-layer ECA attention modules that precisely activate critical local channels, successfully suppressed these background distractions, achieving near-perfect false alarm elimination. Furthermore, in scenarios with dense overlapping crowns (middle column) and edge multi-scale lesions (right column), Edge-Forest YOLO consistently produced bounding box distributions, quantities, and high-confidence predictions that closely matched those of the substantially larger baseline model. In some cases, the bounding box fitting for partially overlapping targets was even more compact.

Overall, the visual analysis confirms the practical engineering value of the task-specific architectural redesign: despite aggressively reducing over half of the redundant parameters, the model maintains intact feature representation. The introduction of attention mechanisms enables substantial suppression of false positives in complex backgrounds, demonstrating clear visual superiority and fully supporting high-reliability, high-precision UAV inspection deployments.

#### 4.5.3 Challenging samples and model limitations

**Figure 12:**
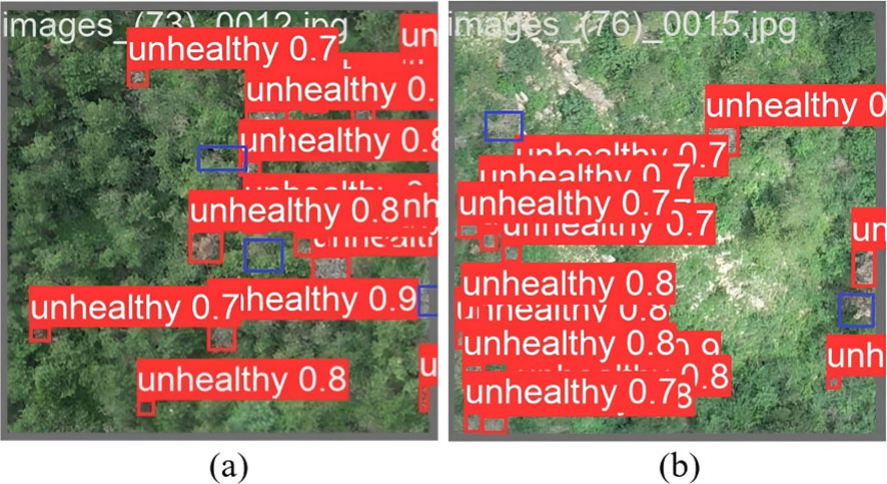
Example of challenges faced by edge-forest YOLO in extreme inspection scenarios; **(A)** early-stage occult lesion interference scenario; **(B)** atypical pathological pattern and complex habitat confusion scenario. Red boxes represent model detection results, and blue boxes represent manually annotated difficult/missed samples.

Although Edge-Forest YOLO achieves a balance between lightweight design and detection accuracy, it is essential to critically examine its performance boundaries under the complex physical conditions of forest inspection. This section focuses on the model’s behavior when handling extreme or challenging samples, as illustrated in **Figure 12**.

The limitations of Edge-Forest YOLO primarily stem from two core challenges:

(1) Visual feature absence in early-stage lesions: As shown in **Figure 12(A)**, when the disease is at a very early stage, the affected pine needles have not yet fully transitioned from green to reddish-brown in their spectral appearance, resulting in textures that are highly similar to healthy crowns. In such extremely low signal-to-noise ratio (SNR) scenarios, the ability of a ultra-lightweight model to capture weak features reaches a physical limit, which partially explains the observed 0.09 miss detection rate.

(2) Confounding by atypical pathological patterns and complex environments: As illustrated in **Figure 12(B)**, in habitats with strong background noise such as exposed rocks or withered grass, lesions exhibiting atypical grayish-brown coloration can easily be misclassified as background while the model attempts to suppress interference. This recognition of inherent limitations demonstrates that, under extreme computational constraints, single RGB modality perception has natural boundaries. These findings provide a clear rationale for future improvements, such as integrating multispectral data fusion or incorporating temporal feature comparison.

## 5 Discussion

### 5.1 Comparison with state-of-the-art methods and advantage analysis

To further clarify the academic and practical value of Edge-Forest YOLO in UAV-based forest disease monitoring, this section conducts a comparative analysis with recent state-of-the-art studies. In the pursuit of high detection accuracy, CPD-YOLO proposed by Yu et al. (2025) and the YOLOv11-based model improved by Shi et al. (2025b) achieved exceptionally high mAPs of 90.42% and 94.7%, respectively. However, their complex architectural designs, such as Transformer-based global modeling, are often accompanied by significant parameter redundancy and computational overhead, making them challenging to deploy on edge platforms with strict resource constraints.

On the other end of the spectrum, in ultra-lightweight deployment scenarios, Edge-Forest YOLO demonstrates significant advantages over comparable edge-oriented models. For instance, LW-PWDNet by Hu et al (Hu and Wang, 2025). reduces the parameter count to 1.9M, achieving an mAP of 89.7%, and Chen et al (Chen et al., 2025). reported a lightweight model with 2.3M parameters and an mAP of 92.8%. In comparison, Edge-Forest YOLO, under an extremely constrained parameter budget of 2.31M, not only achieves ultra-fast inference at 4.2 ms per frame but also maintains a robust mAP@0.5 of 0.927. The key underlying reason is that the proposed non-asymmetric channel reallocation (CR) and cross-layer efficient channel attention (ECA) mechanisms effectively prevent the loss of high-level pathological semantics during network compression, achieving a highly competitive Pareto-optimal trade-off between accuracy and computational cost in the field of forest edge computing.

### 5.2 Limitations and future directions

Despite achieving a desirable balance, Edge-Forest YOLO has certain limitations in real-world, complex environments. Firstly, constrained by the physical boundaries of single-modality RGB vision, the model has limited ability to perceive very early-stage latent lesions. Compared with studies leveraging hyperspectral data to capture subtle early-stage spectral variations (e.g., LE-PWDNet (Shen et al., 2025)), the current model primarily relies on visual discoloration of pine needles, resulting in inherent information loss during the “asymptomatic” phase of the disease. Secondly, the robustness in extreme scenarios still has room for improvement. Under highly dense canopy occlusions or severe motion blur caused by high-speed UAV operations, lightweight spatial feature extraction alone may fail to detect a small fraction of weak semantic targets. Moreover, practical deployment on UAV edge devices may be constrained by battery life, device memory, thermal limits, and communication reliability under dense forest canopies.

To address these limitations, future research will focus on: (1) integrating multispectral remote sensing data and exploring cross-modal feature fusion (e.g., inspired by dual-channel discrete wavelet transform (Zhou and Yang, 2025)) to overcome the limitations of visible-light modalities for early detection; (2) investigating lightweight deblurring preprocessing modules to enhance stability during dynamic inspections. Moreover, practical deployment considerations such as UAV flight endurance, edge device memory and thermal constraints, and communication reliability under dense canopy conditions will be thoroughly evaluated. Finally, the algorithm will be deployed on low-power airborne computing platforms for long-term field trials, aiming to optimize real-time pine wilt disease monitoring workflows and support smart forestry decision-making, early intervention, and disaster mitigation strategies.

## 6 Conclusion

To address the stringent requirements for lightweight modeling and real-time performance in UAV-based pine wilt disease surveillance, this study proposes Edge-Forest YOLO, an ultra-lightweight detection framework built on an improved YOLOv8n. By leveraging scale-aware channel reallocation and asymmetric pruning, the model effectively mitigates computational redundancy in shallow feature maps, reducing the parameter count to 2.31M—a decrease of approximately 23.6% relative to the original YOLOv8n. To compensate for potential accuracy loss from structural slimming, cross-layer efficient channel attention (ECA) and domain-adaptive data augmentation are integrated, resulting in a high detection precision of mAP@0.5 = 0.927 on the validation set. In terms of inference efficiency, Edge-Forest YOLO achieves 4.2 ms per frame, corresponding to 26 FPS on the Jetson Nano edge device, corresponding to an approximate 112% speed-up over the baseline. Visualization analyses indicate that the model maintains robust target discrimination and low false-positive rates, even in complex forest environments with dense occlusion and variable illumination.

Importantly, these results demonstrate that Edge-Forest YOLO not only delivers a favorable trade-off between accuracy and computational efficiency but also enables timely and reliable decision-making in forest health management, supporting early detection, rapid response, and precision intervention in pine wilt disease control. By bridging real-time UAV monitoring with actionable plant health intelligence, the framework provides a scalable tool for operational disease surveillance and data-driven forest management strategies.

## Data Availability

The original contributions presented in the study are included in the article/supplementary material. Further inquiries can be directed to the corresponding author.
